# Predicting myosin heavy chain isoform from postdissection fiber length in human skeletal muscle fibers

**DOI:** 10.1152/ajpcell.00700.2023

**Published:** 2024-01-08

**Authors:** Grace E. Privett, Austin W. Ricci, Julissa Ortiz-Delatorre, Damien M. Callahan

**Affiliations:** Department of Human Physiology, University of Oregon, Eugene, Oregon, United States

**Keywords:** fiber length, MHC, ROC, skeletal muscle

## Abstract

Experimental techniques in single human skeletal muscle cells require manual dissection. Unlike other mammalian species, human skeletal muscle is characterized by a heterogeneous mixture of myosin heavy chain (MHC) isoforms, typically used to define “fiber type,” which profoundly influences cellular function. Therefore, it is beneficial to predict MHC isoform at the time of dissection, facilitating a more balanced fiber-type distribution from a potentially imbalanced sample. Although researchers performing single fiber dissection report predicting fiber-type based on mechanical properties of fibers upon dissection, a rigorous examination of this approach has not been performed. Therefore, we measured normalized fiber length (expressed as a % of the length of the bundle from which the fiber was dissected) in single fibers immediately following dissection. Six hundred sixty-eight individual fibers were dissected from muscle tissue samples from healthy, young adults to assess whether this characteristic could differentiate fibers containing MHC I (“slow” fiber type) or not (“fast” fiber type). Using receiver operator characteristic (ROC) curves, we found that differences in normalized fiber length (114 ± 13%, MHC I; 124 ± 17%, MHC IIA, *P* < 0.01) could be used to predict fiber type with excellent reliability (area under the curve = 0.72). We extended these analyses to include older adults (2 females, 1 male) to demonstrate the durability of this approach in fibers with likely different morphology and mechanical characteristics. We report that MHC isoform expression in human skeletal muscle fibers can be predicted at the time of dissection, regardless of origin.

**NEW & NOTEWORTHY** A priori estimation of myosin heavy chain (MHC) isoform in individual muscle fibers may bias the relative abundance of fiber types in subsequent assessment. Until now, no standardized assessment approach has been proposed to characterize fibers at the time of dissection. We demonstrate an approach based on normalized fiber length that may dramatically bias a sample toward slow twitch (MHC I) or fast twitch (not MHC I) fiber populations.

## Graphical abstract

**Figure:**
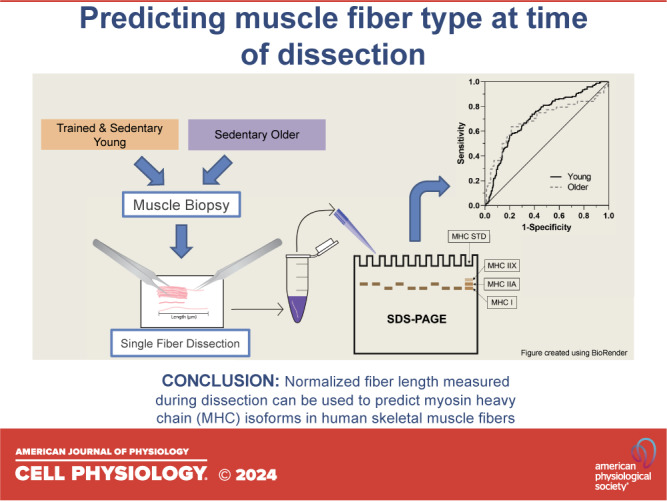

## INTRODUCTION

At the tissue and organismal levels, skeletal muscle function is mediated by the metabolic and contractile properties of the fibers of which they are comprised (1–3). In turn, contractile properties at the fiber level are heavily influenced by the selective expression of distinct myosin heavy chain (MHC) isoforms within each cell. This is due to isoform-specific differences in myofilament protein interactions affecting the rate and duration of myosin/actin cross-bridge formation (4–6). Likewise, metabolic and structural variations across fibers are often associated with differences in MHC isoform expression (6). Therefore, when conducting tissue-level metabolic or proteomic assays, knowledge of the MHC isoform expression in fibers under study is important. In preclinical models, whole muscles are primarily composed of fibers with uniform MHC expression, sometimes leading to a less complicated analysis of outcome measures that are influenced by MHC isoform. For example, investigators may compare assay results from soleus and medial gastrocnemius in the rat to explore the response to intervention in plantar flexor muscles with predominantly MHC I and MHC IIB fibers, respectively (7). However, human muscles are typically heterogeneous in fiber-type distribution within a single whole muscle, complicating the interpretation of results from these assays when they cannot be a priori assigned by MHC isoform. Sodium dodecyl sulfate-polyacrylamide gel electrophoresis (SDS-PAGE) is the gold standard for identifying MHC isoforms when conducting cellular-level contractile assays. However, this method consumes a sample and takes considerable time, thereby precluding the possibility of pre-experimental fiber typing. Although high-throughput and relatively rapid approaches have been recently developed to identify fiber type in single fibers (8), the time required still precludes application in approaches like single fiber contractile mechanics or cellular respiration experiments. Therefore, a method to create a sample of human skeletal muscle fibers with greater fiber-type homogeneity before experimentation would benefit assays influenced by MHC isoform.

During preparation for cellular assays, single muscle fibers are manually dissected from the tissue in which they are contained (“fiber bundle”). Traditionally, fiber dissection involves careful pulling of the fiber, with sharpened forceps, along the plane in which the fibers within the bundle are oriented, thus liberating it from the neighboring fibers. When extracted at 4°C, the tensile stress (parallel to the longitudinal axis) on the fiber during dissection causes the fiber to undergo longitudinal deformation (stretch). However, we and others have anecdotally noted that this stretch is not uniform across fibers and have observed that the degree of stretch is sometimes associated with fiber type. Although it would be logical to assume this variation in dissection-induced stretch depends on fiber stiffness, and/or elastic modulus, the literature is divided as to whether MHC isoform expression influences either of these properties (3, 9). Furthermore, no standard approach for predicting MHC isoform at the time of dissection has been validated. Therefore, we sought to establish the reliability of fiber length, normalized to tissue sample or “bundle” length and measured immediately postdissection, as an estimate of MHC isoform to support its use in preparation for cellular contractile and metabolic assays. To that end, the purpose of this study was to determine if MHC isoform has a significant effect on postdissection fiber length and whether those differences can be used to predict fiber type at the time of dissection, evaluated using receiver operator characteristic (ROC) curves. To explore the impact of chronic modifiers of skeletal muscle stiffness on fiber pull characteristics and subsequent predictive capability of this assay, we applied these tests to samples in which stiffness and morphology were expected to differ based on training status (10) and age (9).

## METHODS

### Subjects

Approval for this study and its procedures was obtained from the Institutional Review Board at the University of Oregon. Participants in this study were recruited from the University of Oregon and the surrounding community as part of other, ongoing studies. Muscle tissue from seven young (19–22 yr, 5 females, 3 males) and 3 older adults (69–75 yr, 2 females, 1 male) were included to evaluate the efficacy of our approach. All participants were healthy by self-report. To eliminate the potential confounding effects of chronic or acute pathology, individuals were excluded for diagnosed chronic illnesses including diabetes, hypertension, neurological or neuromuscular disease, thyroid or other endocrine disease, heart, lung, or kidney disease, autoimmune disease, orthopedic limitation, or known coagulopathy. Potential participants were also excluded if they reported hormone replacement therapy, cancer or treatment for cancer; and/or experienced recent, unexpected weight loss (5 kg bodyweight) within 12 mo. Finally, potential volunteers reporting smoking in the previous 12 mo, or an alcohol or drug use disorder were excluded.

To explore the effect of habitual physical activity on the predictive capability of this assay, we included participants who reported either high habitual physical activity, categorized as “trained” (*n* = 4 young; 2 females, 2 males), or minimal habitual physical activity, categorized as “sedentary” (*n* = 3 young; 2 females, 1 male). “Trained” individuals reported greater than 60 min of structured exercise on at least 5 days per week including resistance training of the lower extremities. “Sedentary” individuals reported no structured exercise and physical activity no more strenuous than brisk walking or cycling. Activity status was confirmed by accelerometry (PAL Technologies Ltd., Glasgow, UK). Samples from four young females and three young males were included in ROC generation and threshold selection for the younger cohort, whereas a sample from a fifth young sedentary female was included only in the ROC application, as a novel sample, for the younger cohort. All older adults met the criteria of our sedentary young cohort and were tested in a separate ROC application to further explore the translation of this approach in separate populations with likely variation in fiber type population, myofiber cross-sectional area, and elastic modulus.

### Muscle Biopsy Procedure

Percutaneous biopsy of the vastus lateralis (VL) muscle was performed by trained laboratory personnel using a sterile technique and under local anesthetic (1% or 2% lidocaine HCL [Hospira Worldwide, Lake Forest, IL]) as previously described (11). To obtain a sample, a 5-mm Bergstrom biopsy needle was inserted through a small incision made in the skin and muscle fascia, and VL muscle was obtained at a depth of ∼2–3 cm.

### Tissue Processing

The collected sample intended for mechanical experimentation was placed in a cold dissecting solution (120.782 mM sodium methane sulfate (NaMS), 5.00 mM EGTA, 0.118 mM CaCl_2_, 1.00 mM MgCl_2_, 5.00 mM ATP-Na_2_H_2_, 0.25 mM KH_2_PO_4_, 20.00 mM 20 *N,N*-bis[2-hydroxyethyl]-2-aminoethanesulfonic acid (BES), 1.789 mM KOH) and parsed into bundles of ∼50 fibers. Bundles were then tied to glass rods, chemically skinned overnight at 4°C, and advanced through solutions of increasing glycerol content (10%, 25%, and 50% glycerol) in 2-h intervals before long-term storage in 50% glycerol solution [5.00 mM EGTA, 2.50 mM MgCl_2_, 2.50 mM ATP-Na_2_H_2_, 10 mM imidazole, 170.00 mM potassium propionate, 1.00 mM sodium azide, EDTA-free protease inhibitor tablet (Thermo Fisher), 50% glycerol by volume] at −20°C until later use. In addition, ∼15 mg of muscle was flash frozen in liquid nitrogen at the time of biopsy for later assessment of MHC distribution via SDS-PAGE.

### Single Fiber Dissection and Measurement

On each day of experimentation, one bundle was chemically skinned (dissecting solution + 1% Triton X-100) at 4°C for 20 min, then placed in dissecting solution at 4°C and trimmed at each end. Bundles were trimmed perpendicular to the long axis of fiber orientation to ensure uniform fiber length through the bundle. Initial bundle length was measured using an in-line mounted camera, attached to a boom-mounted stereoscope, and associated software (Leica Microsystems, Deerfield, IL). Subsequent bundle measurements were made every 15 min or 10 fiber dissections, whichever occurred first. Single fibers were extracted, fiber length was measured within 2 min of dissection, and the fiber was placed into a high-salt sample buffer (2% SDS, 62.5 mM Tris, 10% glycerol, 0.001% bromophenol blue, 5% β-mercaptoethanol, pH 6.8). To normalize fiber length across different fiber bundles, absolute measures (µm) were expressed as a percent of the bundle length from which the fiber was dissected. Samples in buffer were briefly centrifuged, heated for 2 min at 65°C, and stored at −80°C until a later assessment of MHC isoform.

### MHC Isoform Expression

MHC isoforms were subsequently identified in single fibers using SDS-PAGE (12). Briefly, protein extract for each fiber was loaded into its own well of a 4% stacking/7% resolving polyacrylamide gel. For comparison, the MHC standard, prepared with 2.5 mg of tissue multi-fiber homogenate (stored at the time of processing as above) and 95 µL of sample buffer was included at the middle of the gel. Gels were run at 70 V for 3.5 h, followed by 200 V for 20 h at 4°C. Gels were then silver stained, and MHC isoform expression (I, IIA, and/or IIX) was determined by comparison with the MHC standard (see Fig. 3). For the ROC application, relative MHC isoform expression was quantified as integrated density using FIJI software (13).

### ROC Curve Generation

Receiver operating characteristic curves were generated using SPSS statistical software package (IBM, Armonk, NY) to assess the quality of normalized fiber length (% bundle length) as a predictor of MHC isoform. A ROC curve is a plot test that evaluates the performance of a binary classification system. The ROC accounts for sensitivity (Se), the proportion of cases that are correctly predicted as positive, and specificity (Sp), the proportion or cases that are correctly predicted as negative (14). In the current application, the binary classifier was MHC isoform. As such, fibers were classified as either “MHC I” or “not MHC I” (including MHC IIA, IIX, and all hybrid isoforms). The resulting area under the curve (AUC) quantifies the ability of the test variable (in our case, normalized fiber length) to discriminate between the two outcomes [MHC isoform (15)], with a higher value indicating a predictor that successfully balances sensitivity and specificity. Predictive threshold, or cutoff (*c*), values were identified by four previously established approaches enumerated later. Calculations and analysis were performed using MATLAB software (R2020b, The MathWorks, Inc., Natick, MA). A fifth approach, accounting for potential bias toward selection of “not MHC I” fibers due to a high prevalence of MHC II fibers in our samples, was also performed using MATLAB software.

#### Approach 1.

The point closest to the coordinates (*x* = 0, *y* = 1) on the ROC curve is the theoretical optimal cut-off. This point was mathematically identified by solving the following equation to determine the minimum value closest to the point (0,1) (16):

(*1*) $$\text{ER}\left(c\right)=\;\sqrt{{\left(1-\text{Se}\left(c\right)\right)}^{2}+{\left(1-\text{Sp}\left(c\right)\right)}^{2}}.$$

#### Approach 2.

The maximum Youden’s Index (*J*) represents the proportion of correctly predicted positive and negative cases (17). A higher value indicates a stronger predictor. This point was identified as the maximum cut-off value of the equation (16, 18, 19):

(*2*) J(c)=Se(c)−(1−(Sp(c))).

#### Approach 3.

The geometric mean (GM) is an ideal way to assess the performance of a classifier in an imbalanced data set (20). A high GM indicates a strong predictor. To account for a possible effect of imbalanced representation for MHC I and not MHC I fibers, the GM was calculated, and the maximum value was identified:

(*3*) $$\text{GM}=\sqrt{\text{Se}\left(c\right)\times \text{Sp}(c)}.$$

#### Approach 4.

The index of union represents the cut-off point with both maximum sensitivity and specificity (16). This point was calculated as follows, and the minimum value was identified:

(*4*) IU(c)=|Se(c)−AUC|+|Sp(c)−AUC|.

#### Approach 5.

To account for potential bias toward the selection of “not MHC I” fibers, the ROC coordinates corresponding to high specificity (0.900) were identified and indexed to the threshold value.

### Threshold Performance

All identified thresholds were subsequently tested by comparing the threshold-predicted outcome (MHC isoform estimated by cut-off) to the confirmed outcome (MHC isoform identified by SDS-PAGE) for each test variable (normalized postdissection fiber length). The resulting confusion matrices produced a count of true positive (TP) and true negative (TN) outcomes (21), which were used to assess the performance of each threshold by calculation of accuracy, $\frac{\text{TP+TN}}{\text{total no. of fibers}}$, and precision, $\frac{\text{TP}}{\text{no. of predicted positive}}$. Accuracy and precision values range from 0.00 to 1.00, with a value of 1.00 indicating complete agreement between predicted and observed outcomes. To explore the possibility that habitual training and age might affect postdissection fiber length in a way that alters the predictive capacity of generated thresholds, separate ROC curves were generated for young sedentary and trained participants, as well as older adults. Optimal thresholds were identified using the five approaches described previously.

### Threshold Application

To evaluate the efficacy of threshold application on creating bias in single fiber selection, a sample of fibers from a fifth young female (not included in the generation of the ROC thresholds) were dissected at 4°C, measured, and classified as “MHC I” or “not MHC I” based on comparison of postdissection fiber length to a threshold identified from the ROC curve. The threshold determination approach (of the 5 provided) that offered the most conservative cut-off value was selected for this application. Fibers with postdissection lengths shorter than the threshold were classified “MHC I,” and those with postdissection lengths longer than the threshold were classified “not MHC I” and divided into these two groups. Fibers belonging to these groups were collected, flash frozen, and stored at −80°C for later MHC isoform assessment via SDS-PAGE performed collectively on each sample group. The biased homogenates were compared with a standard homogenate from the same subject.

### Statistics

Statistical calculations were performed using SPSS (v.26, IBM, Armonk, NY) with significance set at *P* = 0.05. To account for the effect of varying bundle lengths on extracted fiber length, normalized fiber length was expressed as a percent of the bundle length from which it was dissected (% bundle length). All statistical analyses utilized this normalized fiber length. A linear mixed model, including a random effect to account for potential differences across participants, was run to assess the effect of MHC isoform and training status on postdissection fiber length, with Bonferroni post hoc testing performed for any significant main effect. Given that this assay aimed to differentiate MHC I from all other fiber types (“not MHC I”), statistical comparisons included MHC as a binary variable.

## RESULTS

### MHC Isoform and Postdissection Fiber Length

In younger adults, 25% of the fibers expressed only MHC I isoform and 18% of fibers expressed two or more MHC isoforms (Table 1). During fiber dissection, bundle length was modestly reduced (10.2 ± 11.1%) and accounted for via serial bundle measures. Postdissection fiber length was a significant predictor of MHC isoform (*P* < 0.01), with MHC I fibers producing shorter lengths (114 ± 13%) versus non-MHC I (125 ± 16%) fibers.

**Table 1.** MHC Distribution of skeletal muscle fibers

		I		IIA		IIX		IIA/X	
	*n* Total	*n* (%Total)	Fiber Length (%Bundle)	*n* (%Total)	Fiber Length (%Bundle)	*n* (%Total)	Fiber Length (%Bundle)	*n* (%Total)	Fiber Length (%Bundle)
Young	668	166 (25)	114 ± 13%	311 (47)	125 ± 16%*	72 (11)	122 ± 19%*	113 (17)	125 ± 18%*
Older	194	44 (23)	103 ± 27%	78 (40)	110 ± 18%	10 (5)	122 ± 20%*	54 (28)	122 ± 18%*

Data shown are means ± SD. Data are not shown for myosin heavy chain (MHC) I/IIA fibers due to low representation (13 of 862).

* Significantly different from MHC I fiber length (*P* < 0.01).

### ROC Performance

**Figure 1.**
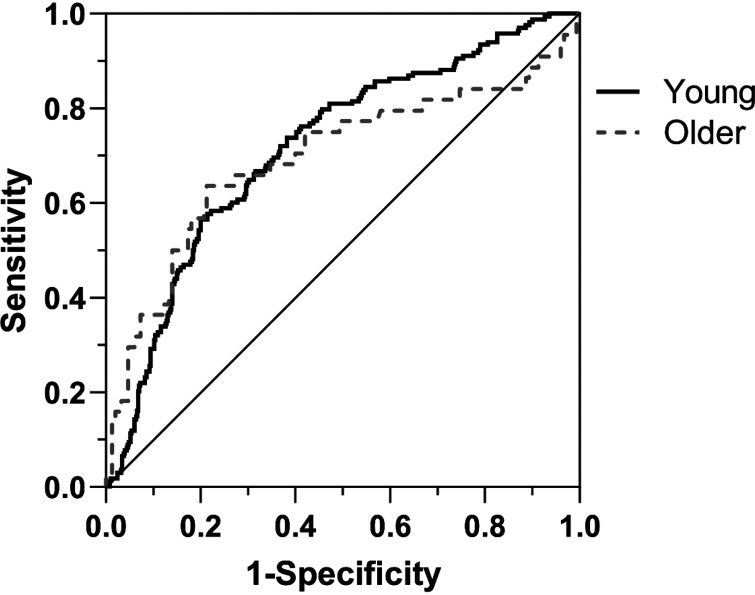
A receiver operator characteristic (ROC) curve evaluating postdissection fiber length as a predictor of myosin heavy chain (MHC) isoform in both young and older adults.

Normalized fiber length and MHC isoform data (confirmed via SDS-PAGE) from 668 fibers in younger adults were included in an ROC curve (Fig. 1), producing an AUC = 0.72. This value is considered excellent by some (21) and fair by others (22). The optimal cut-off values suggested by the ROC in young samples were identified using five approaches, producing threshold values equal to 118% (*approaches 1*, *3*, *4*), 114% (*approach 2*), and 107% bundle length (*approach 5*). The most conservative threshold, 107%, produced the highest values for accuracy (0.75) and precision (0.50), which compares favorably with random sampling, given the heavy bias toward non-MHC I fibers in our sample (see Table 1).

### Assessing the Impact of Habitual Training on Predictive Ability

Of the fibers analyzed from healthy young adults, 56% were from habitually active individuals. MHC I fibers comprised 27% of the trained subset and 22% of the sedentary subset. Normalized postdissection fiber length did not differ by training status (*P* = 0.18). The ROC curve generated from the trained sample produced an AUC = 0.71 and the ROC generated from the sedentary sample produced an AUC = 0.73. The thresholds produced from the trained (106%, 114%, 116%) and sedentary (107%, 122%, 123%) subsets were not appreciably different from those produced from the whole dataset (107%, 114%, 118%). When the most conservative threshold of each subset was tested in a sample of fibers from activity-matched individuals, there was no difference in accuracy (0.74 trained vs. 0.74 sedentary), though precision was greater in trained (0.55) versus sedentary (0.35), likely due to the slightly greater representation of MHC I fibers for trained samples.

### Assessing the Impact of Age on Predictive Ability

**Figure 2.**
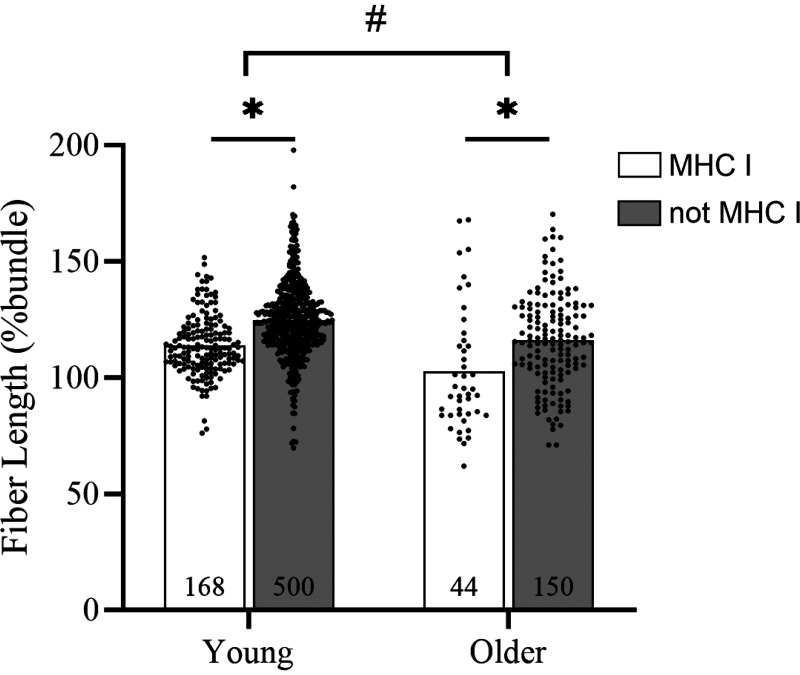
Normalized postdissection fiber length was significantly shorter in myosin heavy chain (MHC) I vs. MHC II fibers (*P* < 0.01) in both young and old adults. In addition, postdissection fiber length was significantly shorter, overall, in sample from older vs. younger adults (*P* < 0.01). There was no significant fiber type by age interaction (*P* = 0.45). Data are shown as mean (bars) with individual points per group/MHC type. *Main effect of fiber type, #main effect of age.

To extend our analysis and establish the durability of our approach in other populations, we repeated our analyses in muscle tissue samples from a cohort of healthy older adults. Of 194 dissected fibers, 44 (23%) were later identified as MHC I. Normalized fiber length measured immediately postdissection differed significantly by fiber type such that MHC I were shorter than not MHC I (Fig. 2; *P* < 0.01). Furthermore, normalized postdissection fiber length was reduced in older adults compared with young overall (Fig. 2; *P* < 0.01). Nevertheless, ROC characteristics suggest a similar capability to discriminate fiber type by normalized length during dissection (Fig. 1). Indeed, AUC generated by fibers from older adults (0.69) was similar to that of the young (0.72) in spite of considerably fewer fibers included for analysis. However, normalized fiber length thresholds produced from fibers of older adults (102% to 106% using *approaches 1*–*4*) were lower than those calculated from young fibers. The most conservative threshold (*approach 5*) was 88% of bundle length, which is again shorter than that prescribed using fibers from younger adults (107%), consistent with the notion that fibers from older adults will have a greater elastic modulus than young (9) and thus resist stretching to a greater degree.

### Attempting to Create a More Homogenous MHC Background during Fiber Dissection

**Figure 3.**
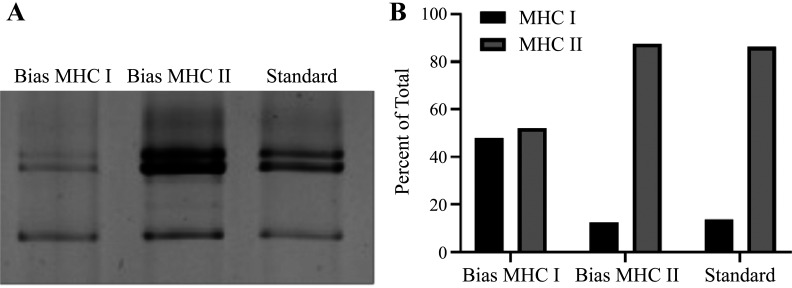
Sodium dodecyl sulfate poly acrylamide gel electrophoresis (SDS-PAGE) results suggested that fiber-type estimation using a length-based threshold allowed for manipulation of the relative fiber-type distribution in each sample (*A*). Quantification of the relative fiber-type distribution in each sample using densitometry confirmed that fiber-type estimation using a length-based threshold enabled the creation of a more even fiber-type distribution (*B*).

As proof of concept, 70 fibers were dissected from the novel sample of younger fibers (not included in the generation of the ROC curve) to test the application of the thresholds identified from the younger ROC curve. Using the most conservative threshold in fibers from younger adults, 107% of bundle length, 32 of the dissected fibers were estimated “MHC I” and 38 fibers were estimated “not MHC I” based on postdissection fiber length <107% of bundle length. Both of these samples, the former biased toward MHC I and the latter biased toward MHC II, were compared with the standard homogenate from the same individual (Fig. 3*A*). The relative isoform distribution of the standard (not biased) homogenate was quantified as 13.6% MHC I, 86.3% not MHC I, and the relative isoform of the sample biased toward MHC II was quantified as 12.4% MHC I, 87.6% not MHC I. The relative isoform distribution of the sample biased toward MHC I was composed of 47.9% MHC I, 52% not MHC I (Fig. 3*B*), demonstrating a substantial shift from the unbiased standard.

## DISCUSSION

### Fibers Behave Differently upon Dissection

Upon mechanical dissection from the prepared bundle, single muscle fibers demonstrate distinct degrees of stretch dependent on MHC isoform expression. This finding, coupled with a strong AUC value, supports the notion that normalized postdissection fiber length serves as a useful predictor of fiber type. We have observed this behavior in skeletal muscle samples from young men and women of varying activity levels and extended our findings to healthy older adults, thus demonstrating its efficacy in a variety of samples that likely contain varying MHC isoform distribution (23), single fiber morphological characteristics (24, 25), and passive viscoelasticity (9). Given the fact that observed behaviors occur well beyond the low end of in vivo temperatures or those maintained during cellular assays, we hesitate to associate stretch and recoil behaviors observed here with fiber type-dependent variations in elastic modulus (26). However, we observe a modest trend toward reduced postdissection fiber lengths in trained versus untrained samples and significant reductions with age, consistent with observations in the literature suggesting exercise training (10, 27) and age (9) enhance elastic modulus in skeletal muscle. Thus, it is logically appealing to assume elastic modulus and resting sarcomere length differ by MHC isoform and this allows the experimenter to dramatically alter the chances of extracting fibers of various isoforms based on the stretch and recoil of a fiber upon dissection. Though one study reported no difference in resting sarcomere length by MHC isoform (28), other work has shown that myosin super-relaxed state, correlated to resting sarcomere length, is dependent on MHC isoform (29). Differences in postdissection fiber length of MHC I versus MHC II fibers may also stem from fiber-type-based differences in cellular passive stiffness. In human skeletal muscle, the association between passive mechanical properties and MHC isoform expression is unclear. One study observed greater elastic modulus, a measure of stiffness that accounts for potential differences in fiber size, in MHC I versus MHC IIA fibers under passive conditions (pCa 8.0) with 2,3-butanedione monoxime (BDM) (3). This difference in elastic modulus could be attributed to differing isoforms of intra- or intermyofibrillar proteins (30–32) or differences in fiber diameter or residual basement membrane (33). However, some groups have observed no difference in single-fiber stiffness across fibers of differing MHC expression (9). It is possible that experimental temperature contributes to this divergence, as some studies are conducted at 25°C (3) whereas others are conducted at 15°C (9). Preliminary data from our laboratory demonstrate no fiber-type difference in passive elastic modulus between MHC I and MHC IIA fibers at 15°C (data not shown). The present assay was conducted at 4°C, which is considerably cooler than the parameters of most mechanical assays. It is possible that this cooler temperature caused increased single fiber stiffness (34) differentially by fiber type. This is speculative but might explain fiber-type differences in elastic recoil that have not been observed in assays conducted at 15°C and above.

### Training Status Does Not Alter Predictive Capability

Training status has been previously linked to cellular-level differences in passive stiffness (10), prompting the notion that habitual training may affect single-fiber pull characteristics during dissection. However, the present results suggest that normalized postdissection fiber lengths are comparable in trained and sedentary participants within a fiber type. Furthermore, predictive thresholds generated by trained and sedentary subsets, and their subsequent accuracy in each subset, suggest that the predictive capacity of this assay is not specific to training status. These findings suggest that this predictive approach can be used to estimate fiber type in samples from trained and untrained participants alike, without modification.

### Age Does Not Alter Predictive Capability

Age has been associated with enhanced passive stiffness in single muscle fibers (9) that is logically related to longitudinal stretch and recoil during the mechanical dissection of single fibers from their native bundle. Indeed, recoil was greater in fibers from older adults than young in our experiments as indicated by postdissection fiber length (Fig. 2). However, the recoil varied by fiber type in ways that allowed separation of MHC isoform by postdissection fiber length (Fig. 1) similar to young. Collectively, our observations suggest that attempts to bias fiber sampling toward the MHC I isoform during dissection simply requires selecting those fibers that recoil to the greatest extent, regardless of the sample origin.

### Normalized Fiber Length Can Be Used to Bias Random Fiber-Type Sorting toward a More Even Distribution

Here, we demonstrate that postdissection fiber length can be used to bias otherwise random selection of single muscle fibers toward a desired distribution of fiber type during dissection. In the present study, the novel sample used to test the ROC threshold was not evenly distributed with regard to fiber type (13.6% MHC I). However, selection based on the ROC-determined threshold for normalized fiber length following dissection resulted in a fiber-type distribution with nearly 50% MHC I fibers. This demonstrates the utility of our approach in significantly influencing the odds that an assay will be composed of MHC I fibers.

### Limitations to the Current Approach

Our proof of concept analyses led us to the conclusion that normalized fiber length varies by MHC isoform in single fibers following dissection, confirming anecdotal reports by our laboratory and others that this characteristic can be used to bias the chances of harvesting MHC I fibers within a mixed sample. However, our sample size is relatively modest and limited to healthy individuals, free from clinical conditions that may impact muscle morphology and function, including cancer cachexia, end-stage osteoarthritis, or multiple sclerosis. Although we are encouraged by the consistent nature of our findings across young trained, young untrained, and older adults, further studies need to be conducted to test whether this approach is equally effective in clinical populations that are often the subject of studies that use single fiber functional assays.

In summary, single skeletal muscle fibers exhibit fiber-specific stretch characteristics when dissected at 4°C. The stretch induced by dissection results in subtle, but significant differences in normalized fiber lengths such that MHC I fibers are shorter than fibers not exhibiting MHC I isoform in healthy young and old, males and females. In our hands, this divergence in postdissection fiber length can effectively be used to estimate fiber type at the time of single fiber dissection and to shift the fiber-type distribution of a sample toward a more even or biased distribution, regardless of the native abundance. This approach may have utility in assays relying on a priori knowledge of MHC background (e.g., cellular mechanical assays or metabolic/proteomic assays).

## GRANTS

The study was supported by Wu Tsai Human Performance Alliance and NIH R21AG077125-01A1.

## DISCLOSURES

No conflicts of interest, financial or otherwise, are declared by the authors.

## AUTHOR CONTRIBUTIONS

G.E.P. and D.M.C. conceived and designed research; G.E.P., A.W.R., and J.O.-D. performed experiments; G.E.P., A.W.R., J.O.-D., and D.M.C. analyzed data; G.E.P., A.W.R., J.O.-D., and D.M.C. interpreted results of experiments; G.E.P. and J.O.-D. prepared figures; G.E.P. drafted manuscript; G.E.P., A.W.R., J.O.-D., and D.M.C. edited and revised manuscript; G.E.P., A.W.R., J.O.-D., and D.M.C. approved final version of manuscript.
